# IMPACT OF A COLLABORATION WITH CLINICAL PHARMACISTS ON OSTEOPOROSIS TREATMENT RATES

**DOI:** 10.1093/geroni/igz038.2610

**Published:** 2019-11-08

**Authors:** Silvina Levis, Violet Lagari, Maribel Garcia, Sue Kwok, Martha Salazar

**Affiliations:** Miami Veterans Affairs Healthcare System, Miami, Florida, United States

## Abstract

Despite the availability of effective drugs to treat osteoporosis, many patients remain untreated and at high risk for developing a fracture. To improve treatment rates, we included clinical pharmacists in the management of patients with osteoporosis. Over 6 months, 30 days after bone mineral density (BMD) results became available to the ordering physician, all patients with an abnormal BMD were evaluated for management according to guidelines if they had not been approached for care. One of three clinical pharmacists discussed patients with the team that included an endocrinologist and a geriatrician. The team made recommendations and pharmacists followed-up by calling patients and prescribing medications. After excluding those who were already on treatment or did not have an indication, 87 patients qualified: 57 (66%) had T-score ≤-2.5, 19 (22%) had osteopenia and high FRAX score, and 11 (12%) osteopenia by BMD and a fragility fracture. After 30 days, the ordering physicians had treated 32/87 (37%) of patients with an indication: 26/57 (46%) patients with T-score ≤-2.5, 1/19 (2%) with high FRAX, and 5/11 (50%) with fractures. After the pharmacists’ intervention, an additional 33/87 (38%) patients were on treatment: 16 with T-score ≤-2.5; 14 with high FRAX, and 3 with fractures; 6 patients were unreachable, 9 declined, and 6 were referred to endocrinology for work-up. By the end of the 6-month period, 75% of patients with an indication received osteoporosis treatment. These results suggest that an osteoporosis intervention employing clinical pharmacists as part of a multidisciplinary team effectively improves osteoporosis treatment rates.

